# Prescription of individual therapeutic exercises via smartphone app for patients suffering from non-specific back pain: A qualitative feasibility and quantitative pilot study

**DOI:** 10.1007/s00508-020-01616-x

**Published:** 2020-02-14

**Authors:** Timothy Hasenöhrl, Thomas Windschnurer, Ronald Dorotka, Clemens Ambrozy, Richard Crevenna

**Affiliations:** 1 Department of Physical Medicine, Rehabilitation and Occupational Medicine, Medical University of Vienna, Waehringer Guertel 18–20, 1090 Vienna, Austria; 2 Orthopaedic City Center-medCLINIC, Dominikanerbastei 3, 1010 Vienna, Austria

**Keywords:** Mobile health, E-health, Individualisation, Acceptance, Communication

## Abstract

**Background:**

The purpose of this qualitative study was the assessment of the feasibility and acceptance of orthopedists prescribing individualized therapeutic exercises via a smartphone app to patients suffering from non-specific back pain.

**Methods:**

A total of 27 patients (mean age 44.8 ± 13.2 years) diagnosed with acute non-specific back pain were prescribed individually chosen therapy exercises via a smartphone app. Before the patients started and after 4 weeks of exercising all participants went through an assessment protocol consisting of questionnaires (Oswestry Disability Index [ODI], Short Form-36 [SF-36], International Physical Activity Questionnaire [IPAQ], Work Ability Index [WAI], Visual Analogue Scale [VAS] back pain, sociodemographic parameters), assessment of functional parameters (handgrip strength, timed up and go test). With 16 randomly chosen patients semi-structured interviews were undertaken at the end of the intervention period. Interview transcripts were analyzed using thematic analysis. Power analysis and a priori sample size calculations were undertaken with the quantitative data.

**Results:**

From the interviews four thematic categories emerged: prior exercise experience, evaluation of exercise intensity, communication with physician via smartphone app, and variability of exercise location. Quantitative analysis of secondary data showed significant improvements in back pain (ODI) as well as quality of life domains “physical functioning”, “bodily pain” and “vitality” (SF-36) of which “bodily pain” was sufficiently powered with the current sample size.

**Conclusion:**

The prescription of therapeutic exercises via smartphone app to patients suffering from non-specific back pain is feasible and well-accepted in patients at all ages. Pilot data additionally pointed towards efficacy of the intervention.

## Introduction

Approximately two thirds of all adults suffer from back pain at least once in their lifetime. Of these, only 15% are diagnosed with a specific structural damage of the spine while the rest are classified as suffering from non-specific back pain [1]. Non-specific back pain is hallmarked by either the absence of or only weak association to a radiologic correlate to the pain symptoms [2, 3]. It has been known for a relatively long time that non-specific back pain might originate from a number of spinal structures including ligaments, facet joints, the vertebral periosteum, the paravertebral musculature and fascia, blood vessels, the annulus fibrosus, and spinal nerve roots, and also from psychological factors, such as stress, dysphoria and depression [1, 4]. Back pain is a massively debilitating disorder affecting functional domains as well as activities of daily living [5]. Moreover, work ability and as a result wealth might be affected by back pain [6, 7]. The current literature shows a highly analgesic effect of physical exercise with particularly strong evidence for strength/resistance as well as coordination/stabilization exercise programs [8]; however, there are health systems where exercise therapy is not easily available to patients because of sparse availability of physical therapists, remote living of patients, or it might be just too expensive. For these patients, utilization of smartphone apps to support carrying out therapy exercises might be a valuable option to receive this kind of treatment. There are a large number of smartphone apps particularly for the self-management of low back pain; however, these apps can only provide recommendations based on screening questions and not on professional medical care [9]. To close this gap, an Austrian company (BHM e‑health services, Vienna, Austria) developed a smartphone app which allows individualized prescription of exercises for non-specific back pain by the treating physician. Moreover, this app (MeineTherapie App®, BHM e-health services, Vienna, Austria) allows communication between the patient and the physician. The primary aim of this study was to find out if this novel method of communication between physicians and patients is feasible and acceptable for patients diagnosed with non-specific back pain. As a secondary aim, quantitative data were collected to identify sensitive parameters which would illustrate the effects of the intervention.

## Patients, material and methods

### Patient population

Patients with back pain who routinely presented at four orthopedic specialist offices located all over Vienna, Austria, and were diagnosed with non-specific back pain were considered eligible for study inclusion. Further inclusion criteria were age between 18 and 69 years, possession of a smartphone, willingness and ability to perform therapeutic exercises following visual and verbal introductions as well as sufficient knowledge of the German language to understand the instructions. Exclusion criteria were any physiological or mental disease which would impair the ability to perform therapeutic exercises, already planned start of an exercise therapy during the intervention period and pregnancy. Sociodemographic characteristics are depicted in Table 1.

**Table 1:** Sociodemographic characteristics of the study sample with differentiation between patients who were interviewed and not interviewed

		Mean ± SD		
Parameter		All patients (*n* = 27)	Interview (*n* = 16)	No interview (*n* = 11)
Age (years)		44.8 ± 13.2	45.1 ± 14.7	44.4 ± 11.3
Education	Compulsory school	*n* = 3 (11%)	*n* = 1 (6%)	*n* = 2 (18%)
	Apprenticeship	*n* = 5 (19%)	*n* = 3 (19%)	*n* = 2 (18%)
	Professional school	*n* = 2 (7%)	*n* = 2 (12%)	*n* = 0 (0%)
	High school	*n* = 10 (37%)	*n* = 7 (44%)	*n* = 3 (27%)
	College/university	*n* = 7 (26%)	*n* = 3 (19%)	*n* = 4 (37%)
Annual family income	€10–25K	*n* = 11 (41%)	*n* = 7 (44%)	*n* = 4 (36%)
	€25–50K	*n* = 14 (52%)	*n* = 8 (50%)	*n* = 6 (55%)
	>€50K	*n* = 2 (7%)	*n* = 1 (6%)	*n* = 1 (9%)
Smoking status	Non-smokers	*n* = 21 (78%)	*n* = 12 (75%)	*n* = 9 (82%)
	Smokers	*n* = 6 (22%)	*n* = 4 (25%)	*n* = 2 (18%)

### Assessments and patient flow

After obtaining written consent, the patients were sent to the Department of Physical Medicine, Rehabilitation and Occupational Medicine of the Medical University of Vienna, Austria, for baseline testing within 1 week after the initial doctor’s visit. After completion of the baseline tests therapeutic exercises which were individually chosen by the treating orthopedist were unlocked for the patients in the respective smartphone app. The patients were now encouraged to perform these exercises independently for 4 weeks and then come back for a follow-up test.

### Outcome measurements

The analysis of the main outcomes, the feasibility and acceptance of the smartphone app, was undertaken via semi-structured interviews following standard guidelines for qualitative research [10]. The interviews were undertaken with 16 patients who were randomly chosen from the whole study sample. The interviews were conducted at the follow-up assessment. They were audio-recorded and transcribed verbatim. After completion of the data acquisition the transcription of the interviews was read independently by two researchers (TH and TW). Identification of categories was undertaken independently by the same researchers and analysis results were merged afterwards. Any ambivalence between the two researchers was solved by discussion.

Quantitative data collection consisted of functional assessments (timed up and go test; handgrip strength, JAMAR®, Patterson Medical, Warrenville, IL, USA), assessment of back pain (Oswestry Disability Index (ODI); visual analogue scale (VAS)), quality of life (SF-12), physical activity (International Physical Activity Questionnaire), work ability (work ability index short form), as well as sociodemographic parameters.

### Smartphone app

The smartphone app which was utilized in this study was developed with the aim not just to provide exercise videos but to be a communication tool between patients and doctors. It was approved as a class 1 medical device by the Austrian Agency for Health and Food Safety (AGES) and registered in the Austrian Medical Devices Registry. The utilized version contained the videos of 48 therapeutic exercises. For each exercise there was one introduction video provided and one to go along while exercising. Depending on the input of the diagnosis based on the Austrian guidelines for acute and chronic non-specific back pain [11] by the treating physician, the app suggested a preset of exercises from the list of exercises. This suggestion could be modified by the physician depending on the estimation of the individual needs of the patient. These specific exercises were then unlocked for the patient. After finishing each exercise, the patients were invited to give feedback via a traffic light system. In case of a “red light” or an excessive number of “yellow lights” the prescribing doctor was alerted and could contact the patient for clarification. If necessary, the list of prescribed exercises could always be modified by the treating physician. Moreover, the physician could always contact each patient to give encouragement, mental support or to unlock new exercises.

### Qualitative data analysis

Data were analyzed using thematic analysis, which involved several steps [10]: the first step involved carefully reading transcripts several times with the aim of identifying participants’ meanings. The second step involved attaching codes to noticeable text segments. The third step involved identification of themes at a wider level and analysis if codes may be combined to form an overarching theme. The final step involved the revision of themes, cross-checking for similarity and overlap as well as distinctions and finally the definition and classification of themes.

Reading of the transcripts was undertaken by two independently working researchers (TH and TW) with the aim to broaden data interpretation. Moreover, this allowed further insight into the emerging themes. The analysis offered is one interpretation of the interviewees’ experiences and it has to be noted that other interpretations are possible. Nevertheless, as the aim was offering a credible and trustworthy interpretation which collects participants’ perceptions and experiences, ‘thick description’ is provided via the use of direct quotations to give the readers the opportunity to evaluate the interpretation themselves.

### Statistics

Quantitative data were compared between baseline and follow-up measurements via dependent t‑tests (IBM SPSS Statistics for Windows, Version 25.0, IBM Corp., Armonk, NY, USA). Post hoc power analysis was calculated with G*Power (Version 3.1.9.4) [12]. Based on the effect size derived from the post hoc power analysis, sample size calculations were undertaken for each outcome parameter. Spearman correlation coefficients were calculated to identify potential associations between back pain and sociodemographic as well as parameters representing physical function.

### Ethical considerations

This study was approved by the ethics committee of the Medical University of Vienna (EK 2233/2017) as well as the ethics committee of the City of Vienna (EK 18-095-0618) and has been performed in accordance with the ethical standards laid down in the 1964 Declaration of Helsinki. All participants gave their informed consent prior to inclusion in the study.

## Results

**Fig. 1:**
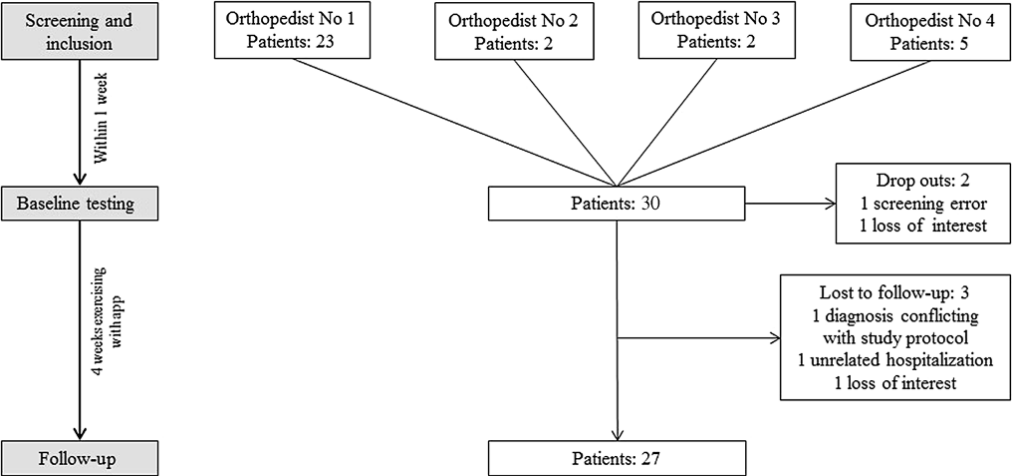
Patient flow and drop-out reasons

A total of 32 patients were included in this study, of whom 27 completed the full study procedure and all measurements. Of the five drop-outs, one patient at the baseline measurement turned out to have been diagnosed with a structural damage of the spine before and was therefore treated as a screening error, one patient was diagnosed with a structural damage of the spine during the test phase unrelated to the exercises, one patient was hospitalized during the test phase due to an unrelated disease, and two patients lost interest to participate in this study, one between screening and baseline measurements and one during the test phase. Patient flow and respective drop-out reasons are depicted in Fig. 1. Of the patients 16 were randomly chosen to go through a semi-structured interview regarding their individual experiences with the app. To assure that the chosen sample was representative for the whole sample, sociodemographic characteristics at baseline between the patients who undertook interviews and those who did not were compared and did not show differences in age, education, family income and smoking status (Table 1).

### Qualitative Analysis

During the thematic analysis four categories were identified: prior exercise experience, valuation of exercise intensity, communication with physician via smartphone app and variability of exercise location.

Pseudonyms are used throughout the manuscript.

#### Prior exercise experience

Patients who were physically active before or were experienced in (back-specific) physical exercises realized this when exercising with the app. They did ot have any problems performing the exercises. Some patients mentioned that prior physical exercise gave them some kind of motor reference value they could use to assess if they performed the current exercises correctly: “I think it’s better to have exercise experience. If someone does not have any exercise experience at all, it will become difficult.” (Carla, aged 60 years). Another patient who reported performing group exercises twice per week phrased it as follows: “Sometimes I was a little bit insecure. But then there was my group exercise which I attend twice per week where I am supervised and getting my feedback. That was helpful because I knew where to be careful. Without this additional training I would have made far more mistakes” (Maria, aged 43 years). Karen, aged 62 years, who was experienced in physiotherapeutic exercises due to an earlier episode of back pain as well as yoga, described that due to her exercise experience she “knew how her body reacts to exercising”. She additionally reported that she already knew many motions and exercise elements from her years of practicing yoga.

Both study patients with and without prior exercise experience consistently performed the app exercises. Moreover, they mentioned problems only occasionally and limited to one or two exercises. The exercise called “Superman” was particularly difficult for several patients. In this exercise the patients had to stand with a forward leaning trunk and raise the arm and contralateral leg at the same time. “I performed all exercises, but there was this one which I had problems with. I mean I did not experience any pain, but it was just very difficult for the muscles. I just could not do it.” (George, aged 43 years). Moreover, some patients experienced performing exercises in a prone position very difficult. Olga, aged 49 years, reported being unable to perform these exercises due to her extreme obesity and Robert, aged 63 years, answered the question why he hated those exercises with, “you are lying prone facing the mat. I do not like that. In the past, I have done some yoga with my wife, but those exercises lying on the belly facing the mat, I will have to say thanks but no thanks. That is because I am having the impression of suffocating.”

Independent of age or exercise experience nearly all study patients reported no fear of doing anything wrong while exercising or that they would have felt safer under the supervision of a therapist. “No, actually I was not frightened at all to do anything wrong. The exercises are not that complicated.” (Robert, aged 63 years). Answering the follow-up question if the presence of a therapist would have increased his perception of safety he answered, “No, I think that is irrelevant. I mean a therapist will surely be in the position to assess more, but I do not think that I had exercises in which that was not possible to do myself.” George, aged 43 years, who described himself as overweight and inexperienced regarding physical exercises answered the same question with, “No, the exercises were not difficult. If I would have felt safer? Probably I would have felt the same.” With a direct reference to the smartphone app Michaela, aged 45 years, said: “Everything was demonstrated well, and the exercises are not those where you would fear that you could hurt yourself”; however, there were two patients, both with no prior exercise experience, who reported that they occasionally felt kind of uncertain with specific exercises and that they would have felt safer under the supervision of a therapist. “When lying on the floor, for example, when they said in the instructional video, ‘extend the head and stretch the spine’, yes you could see it in the video, but if I did it correctly? I don’t know.” (Luis, aged 28 years). Also, one of the older patients said: “I have only one problem, that is when I am lying on the floor, to get back up again”. (Antonia, aged 69 years). It would have been easier for her, if someone would have helped her up after the exercises on the floor.

The question if the patients felt any improvement was answered on two different levels. On the one hand, patients reported the physical improvement: “At first the exercises were rather easy, but then there were some new, strenuous exercises which were particularly exhausting the first few times I had to do them. Over time these exercises became less exhausting and I did not sweat that much or became out of breath that much anymore” (George, aged 43) and, “I can move my head further back now” (Lara, aged 59 years). On the other hand, patients reported an improvement in the management of their exercise protocol: “Because I realized how much time it takes, which object I needed … chair, or whatever equipment I needed to prepare” (Maria, aged 43 years) and, “It became easier because I then already knew the exercises and knew how they worked, but otherwise not.” (Lara, aged 24 years) and, “It became easier over the time course of 4 weeks. You know to bring yourself to do it. That you just do the exercises” (Petra, aged 22 years).

#### Valuation of exercise intensity

The study patients reported that the prescribed exercises were generally not perceived as exhausting; however, there was a high level of heterogeneity regarding the individual perception of the exercises. Some participants reported that the exercises were too easy for them: “I would have thought that the exercises were more exhausting. But with respect to the … ‘aim’ so to say, I think it was actually good because you had to be much more focussed on the exercises themselves. Thus, in my opinion, the primary focus lies on the precision of the exercise execution and to concentrate to the point … the affected muscle, and body region. Actually, when I realized that I nearly had an epiphanic moment.” (Carl, aged 50 years).

Most patients did not realize a visible change in their physical parameters. Some explicitly mentioned that they thought that the exercises were to light for a substantial improvement of their physical performance: “My physical performance did not increase. The exercises were not that exhausting.” (Stefanie, aged 49 years); however, a few patients still reported increased respiration and muscle tone while exercising, and slightly delayed onset muscle soreness was reported infrequently.

The fact that relaxation exercises were part of the smartphone app received particularly positive reception in the study patients independent of prior exercise experience or sex: “Yes, I could have imagined exercising at a higher intensity, but I actually really liked that there was also a focus on relaxation techniques. Because they—and I would not have thought it—were of huge advantage for me. I actually thought that only the strenuous exercises would be beneficial for me but that is not the case. That is not true” (Maria, aged 43 years). Only one patient had a contrary perception regarding the relaxation exercises: “Less relaxation exercises … there were just too many of them” (Lara, aged 24 years).

#### Communication with physician via smartphone app

Generally, messages from the physician with the aim to motivate and encourage the patients were perceived very positively; however, most of the patients would have wanted to have a “re-feedback” system to answer to the messages they received from their physician. “Actually, I would have loved if it would not have been only unidirectional messages but that I could have communicated with the physician. I can imagine that he will not be able to go into every detail when he has got patients in his office, but because I have only those three smileys to rate the exercise, I cannot give detailed feedback which bothers me. Being able to send messages to the doctor could maybe improve this” (Karl, aged 24 years). The aspect of practicability was also mentioned in this context: “It would be great if you could add something to the smileys. I mean, I do not know if this would even work, if he had time to read everything and so on … That’s also the question. Because if you could write something but the doctor does not have time to read it then it is better if this option is not even there” (Robert, aged 63 years). Particularly when there is a problem the option to send a more detailed response would have been helpful: “You are getting a message but you cannot react in any way … One exercise, for example, I ranked only mediocre and received the answer: try again! And then I was insecure and thought: ‘If it might be painful for me, I do not want to do it again. How do I solve this situation?’ Certainly, I could have called the doctor but this is not the intention” (Maria, aged 43 years).

#### Variability of exercise location

Most of the study patients could imagine performing the exercises in various environments and not just only at home. Particularly seated exercises were assessed to being easily carried out at the workplace; however, some limitations were mentioned: “It depends on the exercise. The exercises where I have to lie on the floor certainly not, but maybe those sitting on a chair … You know those exercises where you cannot easily see that you are exercising. I can imagine doing those exercises as a matter of principle” (Lara, aged 24 years) and similarly, “knowing someone could watch me while exercising is no fun” (Robert, aged 63 years). That the particular work environment is essential for the execution at the workplace became obvious: “Neither the premises, nor—I am going to be honest with you—the well-being or whatever you want to call it, would fit … I work in a workshop, a large-capacity workshop with forklift traffic, where it is dirty” (Carl, aged 50 years).

### Quantitative analysis

A total of 27 full datasets were available for quantitative analysis as secondary parameters. The development of the secondary parameters from baseline to the follow-up measurement is shown in Table 2. From all anthropometric parameters, hip circumference was the only one showing a significant change and decreased significantly (−1.54 ± 2.75 cm; *p* = 0.007). Regarding the questionnaires, the ODI decreased significantly (−2.67 ± 4.99; *p* = 0.010) representing diminished back pain. And in the assessment of quality of life, the SF-36 subscales “physical functioning” (+5.0 ± 11.9; *p* = 0.039), “bodily pain” (+14.8 ± 7.8; *p* = 0.000) and “vitality” (+7.2 ± 14.8; *p* = 0.017) increased significantly with higher values representing higher quality of life. Post hoc power analysis of these significant parameters showed the largest effect size in the SF-36 subscale “bodily pain” (Cohen’s dz = 0.744). When transferring this effect size to an “a priori” sample size calculation with the level of significance (α error probability) set at 0.05 and the power (1—β error probability) at 0.80 the required sample size was calculated at 17 participants (Table 3).

**Table 2:** Statistical analysis of the secondary parameters

Parameter	Mean BL	SD	Mean FU	SD	*p*-value	95% CI
*Anthropometric measurements*						
Bodyweight (kg)	81.71	22.53	81.98	22.56	0.333	−0.799 to 0.281
BMI	28.13	7.12	28.20	7.06	0.439	−0.258 to 0.115
Waist circumference (cm)	92.61	17.29	92.32	16.42	0.545	−0.698 to 1.290
Hip circumference (cm)	109.59	14.35	108.06	13.64	0.007	0.450 to 2.625
Waist:hip ratio	0.84	0.09	0.85	0.10	0.061	−0.022 to 0.001
*Functional tests*						
Handgrip strength dominant arm (kg)	35.48	11.26	34.33	12.02	0.194	−0.623 to 2.920
Handgrip strength non-dominant arm (kg)	34.41	10.98	34.19	11.28	0.783	−1.417 to 1.862
Timed up and go test (s)	6.15	1.62	6.00	1.84	0.368	−0.186 to 0.485
Visual analogue scale pain (0–10)	3.22	2.42	3.19	3.11	0.950	−1.172 to 1.246
*Questionnaires*						
Oswestry	17.11	12.98	14.44	13.03	0.010	0.692 to 4.642
IPAQ (MET-Min)	3089.89	2694.69	3722.07	3210.16	0.391	−2121.789 to 857.419
Work ability index	35.28	7.79	38.87	7.84	0.494	−2.350 to 1.165
*SF-36 subscales*						
Physical functioning	72.78	25.05	77.78	23.17	0.039	−9.719 to −0.281
Physical role functioning	50.93	44.66	62.04	39.45	0.117	−25.187 to 2.964
Emotional role functioning	67.90	43.83	80.25	38.41	0.170	−30.317 to 5.626
Social role functioning	80.09	27.13	87.04	17.15	0.126	−15.972 to 2.084
Mental health	71.26	18.10	73.33	20.49	0.522	−8.648 to 4.500
Bodily pain	38.78	16.15	53.59	22.22	0.000	−21.851 to −7.778
Vitality	54.44	21.90	61.67	21.39	0.017	−13.062 to −1.382
General health perceptions	65.15	15.95	68.41	15.49	0.137	−7.630 to 1.111

*MET-min* metabolic equivalent of task minutes

**Table 3:** Post hoc power analysis and a priori sample size calculation of the statistically different outcome parameters

Post hoc effect size and power						Sample size calculation	
	α‑error probability	Total sample size	Non-centrality parameter δ	Effect size	Power (1—β error probability)	Power (1—β error probability)	Required sample size
Oswestry	0.05	27	1.067	0.205	0.177	0.8	189
SF-36 physical functioning	0.05	27	1.075	0.207	0.179	0.8	186
SF-36 bodily pain	0.05	27	3.869	0.745	0.961	0.8	17
SF-36 vitality	0.05	27	1.734	0.334	0.386	0.8	73

### Correlations

Several significant correlations between back pain (ODI) and other parameters were noted of which the clinically relevant ones are depicted here and in detail in Table 4. Back pain showed a strong inverse association with working ability at all time points. Moreover, back pain showed a moderate to strong inverse correlation with handgrip strength of the dominant and non-dominant side also at all time points. While back pain moderately correlated with age at baseline, this association was not significant any more at the follow-up and while the education level was moderately associated with work ability at baseline, it was not significant any more at the follow-up measurement (Table 4).

**Table 4:** Correlations of Oswestry scores and education with age, handgrip strength and work ability index

			Age	Handgrip strength dominant BL	Handgrip strength non-dominant BL	Handgrip strength dominant FU	Handgrip strength non-dominant FU	Work ability index BL	Work ability index FU
Spearman Rho	Oswestry BL	Correlation coefficient	0.402	−0.515	−0.473	−0.622	−0.649	−0.764	−0.834
		Sig. (2-sided)	0.037*	0.006*	0.013*	0.001*	0.000*	0.000*	0.000*
		N	27	27	27	27	27	27	27
	Oswestry FU	Correlation coefficient	0.330	−0.421	−0.381	−0.533	−0.562	−0.647	−0.813
		Sig. (2-sided)	0.093	0.029*	0.050*	0.004*	0.002*	0.000*	0.000*
		N	27	27	27	27	27	27	27
	Education level	Correlation coefficient	n.a.	n.a.	n.a.	n.a.	n.a.	0.479	0.364
		Sig. (2-sided)	–	–	–	–	–	0.012*	0.062
		N	–	–	–	–	–	27	27

*p < 0.05; *n.a.* not applicable

## Discussion

Back pain is not just a debilitating condition for patients but also a substantial burden for national economies [13, 14]. The fact that the majority of the patients are diagnosed with non-specific back pain increases the necessity for exercise therapy [15], which might be difficult to receive under specific circumstances. The amount of care needed for supervised exercise therapy and hence its cost might be a financial obstacle for either health care systems where this kind of therapy is covered by government health insurance, or in countries where health care does not cover the expenses for exercise therapy, for the patients personally. Group exercises might be a possible approach to this; however, individual care is practically impossible in this setting. So, another possibility to answer this problem is the use of mobile health devices (mHealth). There is a plethora of commercially available smartphone applications which provide patients suffering from back pain with exercises [9]; however, if those apps even have individual exercise prescription, then it is limited to the results of screening questions at the start of the app use, which is very limited regarding the specificity of the exercise selection. Chhabra et al. (2018) [16] approached this problem and showed that when physicians used a smartphone app to lead their patients through an exercise program it was effective particularly regarding disability; however, after the initial consultation their patients did not have any interaction with their doctors anymore and the exercises were not specifically designed for back pain. The smartphone app assessed in the present study tried to approach all of the above problems by creating a communication interface between patient and physician and by providing specific exercises for back pain. To our knowledge this is a novel approach in the utilization of smartphone apps for the treatment of patients diagnosed with non-specific back pain.

Mobile health devices (mHealth) have been on the rise as tools for the self-management in various diseases and have been currently discussed controversially regarding their feasibility and efficacy [9, 16, 17]. The results of the semi-structured interviews showed clearly that this smartphone app was feasible and acceptable for the patients. Moreover, analysis of secondary parameters indicated efficacy particularly well in patient-reported outcomes. These are essential in patient-centered care, especially in non-pharmacological interventions for back pain, as the outcomes are directly related to the motivation of the patients [18]; however, regarding the outcome assessment “back pain” the results indicate that measuring back pain with the ODI seemed to have been more sensitive in this patient population than the assessment with the VAS. Similar advantages of the ODI have been shown regarding mobility [19]. This seems logical, as the ODI allows a more complex assessment of the impairment for activities of daily living due to back pain, while the VAS only assesses the pain at the specific assessment time points.

Regarding the correlations, two details caught the eye from a clinical perspective. First, age was associated with back pain at baseline but not at the follow-up anymore. It therefore seems as if the therapeutic exercises were able to diminish the effects of age on back pain. This is in line with the current literature which showed that exercise alone can reduce the risk of back pain [20]. Second, work ability was associated with education at baseline but not at the follow-up anymore. Under the consideration of the association between low education and physical work this is logical at baseline and showed that this seems to be diminished by exercising at the follow-up. This potential association has already been discussed earlier [21]. Moreover, that tailored physical activity is beneficial for the work ability has been shown before [22]. The present data confirmed that this positive effect also seems to be present when the tailored exercise program is provided via an interactive smartphone app. This is particularly relevant, as in the field of exercise intervention in back pain, there is currently no other smartphone app which allows communication between patient and physician [9]; however, results from research analyzing smartphone apps developed for diabetes patients indicate that communication between patient and physician via app might be an important factor for the efficacy of the intervention [23]. This might have been a similar factor in this study.

As in any study, this one has limitations as well. Interviews were not undertaken with all participants but only with about half of the included patients; however, as it had been shown that sociodemographic characteristics between the patients undertaking interviews and those who did not were similar at baseline, this is negligible. Moreover, a larger sample size would have been desirable; however, due to the qualitative study design the primary aim of this study regarding feasibility and acceptance could be clarified conclusively, while the post hoc power analysis and sample size calculation clearly showed the distinctive strengths and limitations of the quantitative results.

The results of the qualitative analysis showed that it was feasible and well accepted in a typical patient population. Moreover, quantitative secondary data, which was initially collected for power analysis purposes only, already showed significant improvements in back pain and quality of life. This is in line with previous literature [24], which showed beneficial effects of exercise on trunk flexibility [25, 26], perceived disability and functionality [27, 28], and back pain symptoms [24, 27]. The post hoc power analysis even showed that the SF-36 subscale “bodily pain” was sufficiently powered with the current sample size which means that the pain-related quality of life improved significantly over the exercise period.

## Conclusion

Specialist prescription of a tailored therapeutic exercise program via a smartphone app for patients suffering from non-specific back pain is feasible and well accepted. Pilot data additionally point towards efficacy of the intervention.
